# Pan-cancer analysis connects tumor matrisome to immune response

**DOI:** 10.1038/s41698-019-0087-0

**Published:** 2019-05-22

**Authors:** Su Bin Lim, Melvin Lee Kiang Chua, Joe Poh Sheng Yeong, Swee Jin Tan, Wan-Teck Lim, Chwee Teck Lim

**Affiliations:** 1 NUS Graduate School for Integrative Sciences & Engineering, National University of Singapore, Singapore, Singapore; 2 Department of Biomedical Engineering, National University of Singapore, Singapore, Singapore; 3 Division of Radiation Oncology, National Cancer Centre Singapore, Singapore, Singapore; 4 Division of Medical Sciences, National Cancer Centre Singapore, Singapore, Singapore; 5 Oncology Academic Clinical Programme, Duke-NUS Medical School, Singapore, Singapore; 6 Department of Anatomical Pathology, Singapore General Hospital, Singapore, Singapore; 7 Institute of Molecular and Cell Biology, Agency for Science, Technology and Research, Singapore, Singapore; 8 Sysmex Asia Pacific Pte Ltd, Singapore, Singapore; 9 Division of Medical Oncology, National Cancer Centre Singapore, Singapore, Singapore; 10 Office of Clinical Sciences, Duke-NUS Medical School, Singapore, Singapore; 11 Mechanobiology Institute, National University of Singapore, Singapore, Singapore; 12 Institute for Health Innovation and Technology, National University of Singapore, Singapore, Singapore

**Keywords:** Cancer genomics, Predictive markers

## Abstract

Recent sequencing efforts unveil genomic landscapes of tumor microenvironment. A key compartment in this *niche* is the extracellular matrix (ECM) and its related components – matrisome. Yet, little is known about the extent to which matrisome pattern is conserved in progressive tumors across diverse cancer types. Using integrative genomic approaches, we conducted multi-platform assessment of a measure of deregulated matrisome associated with tumor progression, termed as tumor matrisome index (TMI), in over 30,000 patient-derived samples. Combined quantitative analyses of genomics and proteomics reveal that TMI is closely associated with mutational load, tumor pathology, and predicts survival across different malignancies. Interestingly, we observed an enrichment of specific tumor-infiltrating immune cell populations, along with signatures predictive of resistance to immune checkpoint blockade immunotherapy, and clinically targetable immune checkpoints in TMI_high_ tumors. B7-H3 emerged as a particularly promising target for anti-tumor immunity in these tumors. Here, we show that matrisomal abnormalities could represent a potential clinically useful biomarker for prognostication and prediction of immunotherapy response.

## Introduction

The extracellular matrix (ECM) is a complex multi-spatial meshwork of macromolecules with structural, biochemical and biomechanical cues, influencing virtually all fundamental aspects of cell biology.^1^ Although tightly controlled during normal development, ECM is frequently altered in many diseases, including cancer.^2,3^ Despite clear evidence of abnormal tumor matrix in cancer, characterization and understanding their functional role in tumors have been challenging, possibly owing to complex nature of ECM proteins and their associated factors, or matrisome.^4–6^ Little is known about the extent to which matrisome pattern is shared across various carcinomas or unique in tumors of differing metastatic potential; it remains unknown as to whether there exist subclasses of tumor matrisome that modulates tumor initiation and response to therapy, particularly in the context of immune response.

Tumor matrisome index (TMI) is a 29-matrisome-gene expression based classifier that has been validated for its predictive value in prognosis and adjuvant therapy response in early-stage non-small cell lung cancer (NSCLC).^7,8^ TMI comprises exclusively of a small set of matrisome gene signatures, primarily encoding non-core matrisome proteins, which were found to be most differentially expressed in NSCLC relative to matched normal epithelium. In essence, TMI is calculated by the sum of the expression level that is multiplied by predefined Cox proportional hazards model coefficient of each TMI gene (see Methods).

Here we hypothesized that this specific pattern of deregulated matrisome could be a common determinant of tumor aggression irrespective of tumor origin. Given the prognostic significance of TMI in predicting the response to adjuvant chemotherapy (ACT) in NSCLC,^8^ we investigated if TMI would be associated with signatures predictive of clinical response to immune checkpoint inhibitor (ICI) treatments, including total mutational burden (TMB), PD-L1 expression, tumor-infiltrating lymphocytes (TILs), and immune gene signatures.^9^ Through parallel analyses of whole-transcriptomic and matched proteomic data, we report robust associations of TMI with TMB, histopathological and clinical features, and reveal the immune landscape of matrisome-deregulated tumors. Our resource of curated compendiums of 8,386 genome-wide profiles, and molecular and clinical associations of TMI may advance our understanding on the underlying role of biophysical matrix changes that is common across cancer types.

## Results

### Matrisome is commonly deregulated in human cancers

**Fig. 1:**
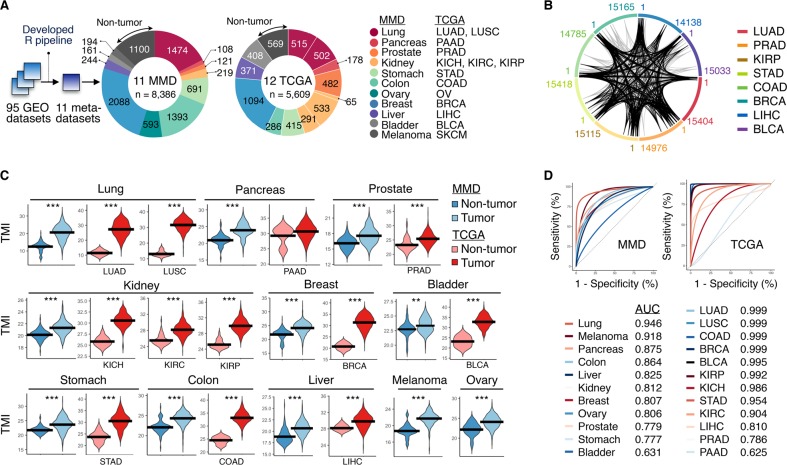
Common matrisome variation in human cancers. **a** Schematic of the study design: 11 cancer type-specific, merged microarray-acquired dataset (MMD) were newly generated for parallel analyses with TCGA cohorts. **b** Circular plot illustrating the ranked position of matrisome genes based on differential expression (cancer vs. normal) in TCGA cohorts. 1 denotes the most differentially expressed gene (DEG). Black and gray lines represent the ranked position of *generic* and lung-specific TMI signature, respectively. **c** TMI in tumor vs. non-tumor tissues across 11 cancer types. The black horizontal line indicates the mean of the samples. ***Mann–Whitney U-test *P* < 0.001, **P < 0.01, *P < 0.05. **d** Area under the ROC curves (AUCs) of the TMI classifier for all cancer types. Smooth ROC curves are drawn for MMDs (left) and TCGA cohorts (right)

The present study was conducted to extend the investigation of TMI performance into 11 major cancer types (Fig. 1a). To facilitate multi-platform parallel analyses with RNA-seq-acquired TCGA data, we first generated a unified, cancer type-specific, merged microarray-acquired dataset (MMD), comprising 8386 transcriptome profiles of tumor and tumor-free tissues, using our previously developed R pipeline^7^ (Supplementary Data files S1 and S2; see Methods). As TMI biomarkers were derived from lung cancer, we first queried the extent to which genome-wide expression patterns of lung cancer would be shared across various cancers.

Using MMD and TCGA, we found that the differential expression patterns of lung cancers, relative to non-tumor controls, were comparable to that of breast, ovarian, bladder, colorectal, and prostate cancers, regardless of assayed platforms (Tables S1 and S2), as recently observed in systematic pan-cancer analyses.^10,11^ Several of the 29 TMI genes were consistently placed in the top ranked differentially expressed gene (DEG) list across multiple cancer types at the individual gene level (Fig. 1b and Table S3). We identified a subset of genes that was significantly enriched in lung adenocarcinoma (UAD), prostate adenocarcinoma (PRAD), kidney renal papillary cell carcinoma (KIRP), stomach adenocarcinoma (STAD), colon adenocarcinoma (COAD), breast invasive carcinoma (BRCA), liver hepatocellular carcinoma (LIHC) and bladder urothelial carcinoma (BLCA), and we termed it as a *generic* TMI signature (Supplementary Data file S3; see Methods). This initial screen supported our hypothesis that altered matrisome dynamics might represent a common phenotype across different malignancies.

### TMI distinguishes cancers from normal tissues

Except for TCGA PAAD (pancreatic adenocarcinoma), which had an insufficient number of normal samples (*n* = 4), all cancer types demonstrated significant difference in the TMI between tumor and non-tumor tissues (Fig. 1c and Supplementary Data files S1, S4 and S5). Expectedly, NSCLC-derived TMI achieved near-perfect diagnostic accuracy in lung cancer datasets, where the area under the receiver operating characteristic (ROC) curve (AUC) at the optimal cut-offs was 0.946, 0.999, and 0.999 in lung MMD, TCGA LUAD, and LUSC (lung squamous cell carcinoma), respectively (Fig. 1d). Across all malignancies, we observed an overall classification accuracies of 82% and 92% using MMD and TCGA datasets, respectively (Table S4). Of note, the observed difference between the two assayed platforms (MMD vs. TCGA) is possibly due to different number of TMI genes filtered in TCGA for final index computation (Supplementary Data file S2; see Methods).

To perform a pan-cancer analysis with minimal technical variability across samples, we next analyzed an independent data source in which tumors were procured under standard conditions (Fig. S1 and Supplementary Data file S6; see Methods). Although over a narrower range than for lung cancer, 1492 carcinomas spanning 10 cancer types displayed a wide and diverse TMI distribution, suggesting a varying degree of matrisome abnormalities at different stages of cancer progression and a specific TMI spectrum for each cancer type.

### TMI is predictive of patient survival

**Fig. 2:**
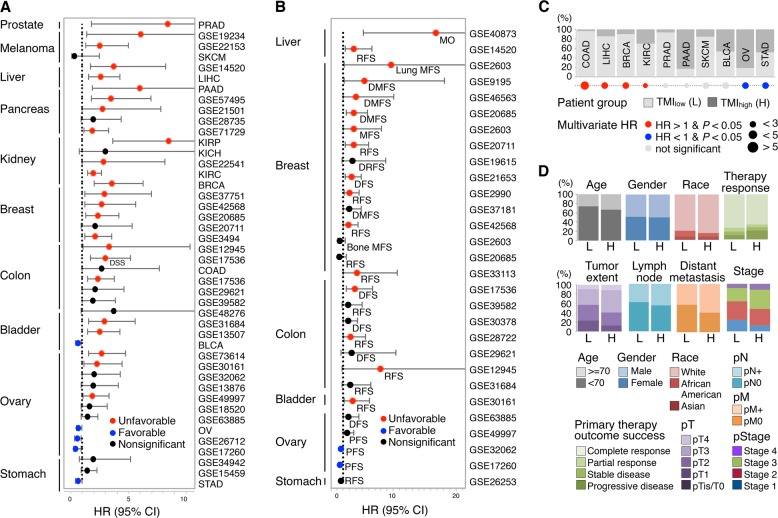
Clinical outcomes in correlation with TMI. **a** Hazard ratio (HR) forest plot for overall survival (OS) and disease-specific survival (DSS) endpoints (total sample size = 8957). **b** HR forest plot for other survival endpoints (total sample size = 4502). Abbreviated names of survival endpoints are provided in Supplementary Data file S7. **c** Patient stratification based on predefined cut-offs and multivariable HR of each TCGA dataset. **d** Comparison of conventional clinical parameters between TMI_low_ (L) and TMI_high_ (H) patients

We obtained a total of 72 independent validation cohorts annotated with clinical information, including overall survival (OS), disease-specific survival (DSS), disease-free survival (DFS), relapse-free survival (RFS), metastasis-free survival (MFS), and/or progression-free survival (PFS) data. As previously described,^8^ a cut-off index was determined for each dataset to stratify patients into TMI_low_ and TMI_high_ groups. Univariable survival analyses revealed a cancer-specific association of TMI in predicting time to death, recurrence, and distant metastasis (Fig. 2a, b; Supplementary Data file S7; see our earlier work for lung cancer^8^). TMI_high_ was an unfavorable prognostic factor for OS in colon, liver, renal, and breast cancers, whereas it appeared to confer a favorable prognosis in ovarian and gastric cancers, even after adjustment for clinical parameters on multivariable analyses using TCGA datasets (Fig. 2c and Supplementary Data file S8). Comparing with traditional clinical features between TMI_low_ and TMI_high_ patients from TCGA datasets, we found that the TMI_high_ group had a higher proportion of patients staged pathologically as T3 or T4, diagnosed as lymph node and distant metastasis positive, and classified as late stage (Fig. 2d).

### TMI is associated with pathological and molecular features

**Fig. 3:**
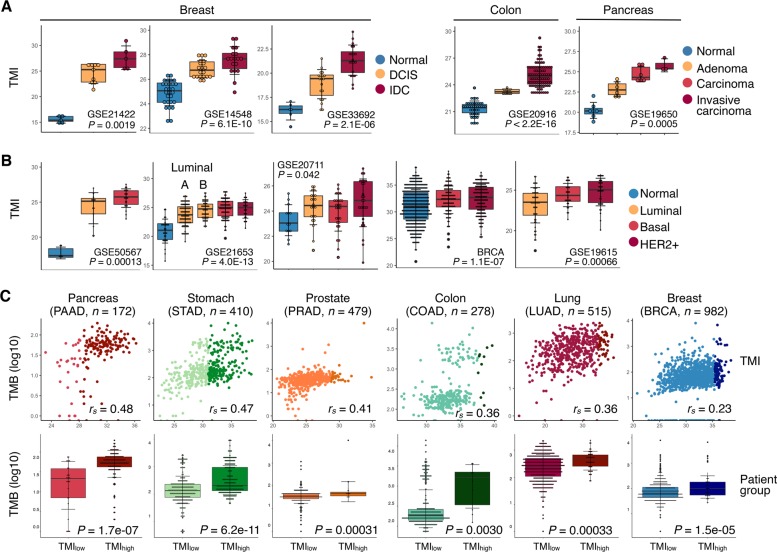
Tumor pathology and molecular features associated with TMI. **a** Tumor pathology associations with TMI in breast, colon and pancreas cancers. **b** TMI stratified by molecular subtypes in breast cancer. **c** Correlation of TMI with total mutational burden (TMB) in TCGA cohorts. Patient samples in each dataset are stratified into TMI_low_ or TMI_high_ group based on the optimal predefined TMI cut-offs. Linear regression lines are drawn (black line) with 95% CI (gray zone); *n* = number of samples analyzed; *r*_*s*_ = Spearman’s correlation coefficient; Mann–Whitney U-test *P*-values are stated. For **a** and **b**, Kruskal–Wallis *P*-values are stated. For **a**–**c**, box hinges represent 1st and 3rd quartiles, and middle represents the median. The upper and lower whiskers extend from hinges up and down indicate the most extreme values that are within 1.5*IQR (interquartile range) of the respective hinge. The short horizontal lines represent the standard deviations

We next asked if there would be a change in TMI during multistep carcinogenesis. In breast, colorectal, and pancreatic cancers, we observed increasing TMI values corresponding to the progressive steps of oncogenesis (normal to adenoma to carcinoma in-situ to invasive carcinoma; Fig. 3a and Supplementary Data file S9). Next, we further investigated for an association between TMI and known prognostic molecular phenotypes in breast cancers (Luminal A/B, HER2+ and basal subtypes).^12,13^ Our prognostic TMI was highest among the most adverse molecular phenotypes (basal and HER2+ tumors), which were known to harbor the worst prognosis (Fig. 3b and Supplementary Data file S10).

We also explored if TMI expression would be correlated with genomic mutational burden, and analyzed matched whole-exome sequencing-derived data of nine TCGA cohorts (pancreas, stomach, prostate, colon, lung, breast, liver, kidney, and bladder), for which TMI has previously been computed (Table S5). TMI was positively correlated with somatic TMB across cancer types (Spearman’s rho test *r*_*s*_ = 0.23 and *P* = 1.77E-14 (Breast) to *r*_*s*_ = 0.48 and *P* = 1.41E−11 (Pancreas); Fig. 3c, top; Supplementary Data file S11). Additionally, based on a previously defined cut-off for discretization into TMI_high_ and TMI_low_, the former harbored significantly higher TMB in all cancers (Fig. 3c, bottom), except for renal (Mann–Whitney U-test *P* = 0.104) and liver cancers (Mann–Whitney U-test *P* = 0.065).

### TMI in the context of immune response

**Fig. 4:**
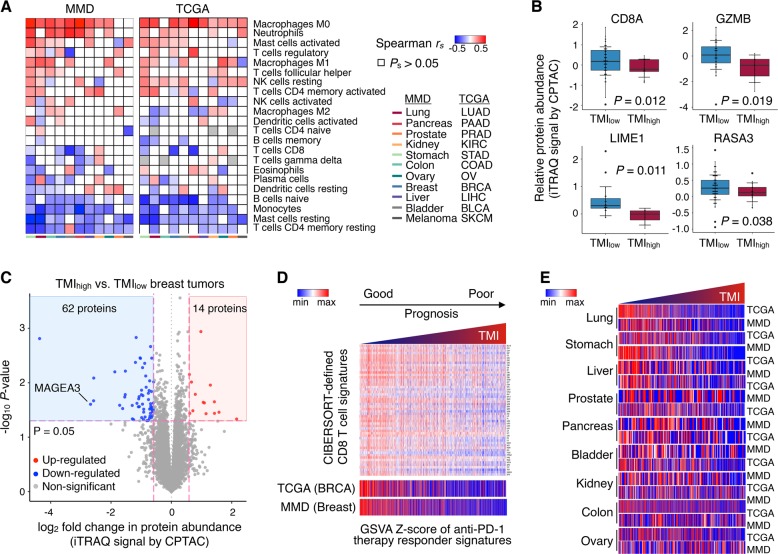
TMI in the context of immune response. **a** Heatmaps showing Spearman’s correlations between TMI and the relative abundance of 22 immune cell types estimated by CIBERSORT for 11 cancer types in MMD (left) and TCGA (right). Spearman’s correlation coefficients and *P*-value are denoted as *r*_*s*_ and *P*_*s*_, respectively. Columns and rows are ordered by increasing number of correlations with statistical significance found in each dataset and immune cell type, respectively. **b** Relative protein abundance of CIBERSORT-defined CD8 T cell signatures in TMI_low_ vs. TMI_high_ breast tumors (TCGA BRCA). One-tailed t test *P*-values are stated. Box hinges represent 1st and 3rd quartiles, and middle represents the median. The upper and lower whiskers extend from hinges up and down indicate the most extreme values that are within 1.5*IQR (interquartile range) of the respective hinge. The short horizontal lines represent the standard deviations. **c** Volcano plot depicting differentially expressed proteins in the two groups stratified by TMI in TCGA BRCA cohort. Red dots represent proteins with fold change (FC) > 1.5 and *limma P* < 0.05; Blue dots represent proteins with FC < -1.5 and *limma P* < 0.05; Gray dots represent proteins with either −1.5 < FC < 1.5 or *limma* P > 0.05. **d** Heatmap showing gene expression of CIBERSORT-defined CD8 T cell signatures (top). Heatmaps showing GSVA z-score of the anti-PD-1 immunotherapy responders’ signatures (IPRES) in breast cancer (bottom) and **e** nine other cancer types using MMD and TCGA datasets. Columns are ordered by increasing TMI

Given the evidence in support of TMB as a robust determinant of tumor immunogenicity in many solid tumors,^14,15^ and that TMI is closely associated with TMB, we next asked if there would be any direct association of this specified matrisomal pattern with the cancer immune landscape. Applying machine learning-based CIBERSORT to MMD and TCGA cancer patient samples (see Methods), we observed a close correlation between TMI and the composition of specific TIL populations for several cancers (Fig. 4a). Enrichment of these TILs related to both innate and adaptive immunity was diverse and cancer type-specific. Relative abundance of M0 and M1 macrophages, neutrophils, activated mast cells, regulatory T cells (Treg), and T follicular helper (Tfh) cells, activated CD4+ memory T cells increased, while resting CD4+ memory T cells, mast cells, naive B cells, and resting dendritic cells decreased with tumor-promoting matrisomal change (Supplementary Data file S12). Impact on immune infiltrates was particularly pronounced in gastric, lung, colorectal and breast cancers, having significant positive and negative correlations with TMI, thus highlighting the influence on the immune contexture during matrisome remodeling in this subset of cancers.

Early works suggest that signatures of T cell states, particularly that of CD8+ T cells, may predict clinical response to ICI-based immunotherapy.^16–19^ Of all cancer types analyzed, breast (BRCA) and pancreatic (PAAD) cancers demonstrated pronounced negative correlation with the estimated abundance of CD8+ T cells, indicating that TMI_low_ tumors harbored higher CD8+ T-cell infiltration levels in these cancers. To validate their differential expression at the protein level, we assessed matched proteomes of TCGA samples provided by the NCI Clinical Proteomic Tumor Analysis Consortium for breast (BRCA) cancers, for which samples were previously classified as either TMI_low_ or TMI_high_ at the transcriptomic level (CPTAC; see Methods).

A matched-cohort assessment of 108 BRCA samples revealed higher protein levels of CIBERSORT-defined CD8+ T cell signatures, including CD8A, GZMB, LIME1, and RASA3, in TMI_low_ tumors (Fig. 4b). Interestingly, differential expression analysis of whole proteomes comparing the two groups (TMI_high_ vs. TMI_low_) identified MAGEA3, a frequently expressed tumor-specific antigen, as the second most highly expressed protein in TMI_low_ tumors (Fig. 4c). The functional contribution of spontaneously occurring MAGEA3-reactive CD8+ T cells to favorable prognosis^20^ may explain the better patient outcomes consistently observed in TMI_low_ breast tumors.

Abundance of CD8+ T cells has been associated with better response to immunotherapies.^9,17,18^ Given the enriched CD8+ T cell signatures at both transcript and protein levels in TMI_low_ tumors, we next investigated the association between TMI and recent reported predictive signatures for immunotherapy response. The *responder* signature comprises 161 genes, which were highly expressed in anti-PD-1 responding melanoma patients compared to non-responding patients^21^ (see Methods). Intriguingly, TMI was highly negatively correlated with the gene set variation analysis (GSVA) z-scores of the *responder* signature for each breast cancer sample (TCGA BRCA; see Methods); higher levels of GSVA z-scores of the *responder* signature were found in TMI_low_ tumors, in which CIBERSORT-defined CD8+ T cell signatures were enriched in these selected tumors (Fig. 4d). Extending the analysis to the other cancer types, we found that except for melanomas, TMI had negative correlations between the two variables, to different extents, with the most pronounced association seen in lung cancer (Fig. 4e).

### B7-H3 as a potential immune target for TMI_high_ tumors

**Fig. 5:**
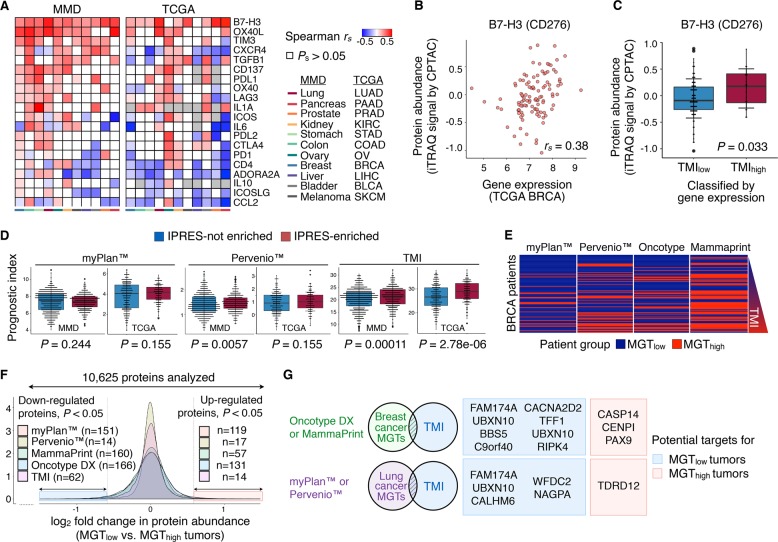
B7-H3 as a promising pan-tumor immune target for TMI_high_ tumors. **a** Heatmaps showing Spearman’s correlations between TMI and gene expression of 20 clinically targetable immune checkpoints for 11 cancer types in MMD (left) and TCGA (right) cohorts. Spearman’s correlation coefficients and *P*-value are denoted as *r*_*s*_ and *P*_*s*_, respectively. **b** Correlation between CD276 (B7-H3) gene expression and protein abundance using TCGA BRCA patient samples; *r*_*s*_ = Spearman’s correlation coefficient, Spearman’s *P*_s_ < 0.05. **c** Relative B7-H3 protein level (iTRAQ signal by CPTAC) in TMI_low_ and TMI_high_ breast tumors (TCGA BRCA); one-tailed t test *P*-values are stated. **d** Prognostic indices in *IPRES-enriched* vs. the rest of lung cancer patients based on three lung cancer-derived MGTs; Mann–Whitney–Wilcoxon test *P*-values are stated. **e** Patient stratification based on two lung cancer-derived MGTs (myplan^TM^ and Pervenio^TM^) and two breast cancer-derived MGTs (Oncotype DX and MammaPrint) in relation with TMI. Rows are ordered by increasing TMI. **f** Distribution of 10,625 proteins in MGT_high_ vs. MGT_low_ tumors with a ± 1.5-fold expression change cut-off based on the *limma* analysis. **g** Venn diagram showing overlapping differentially expressed gene signatures upregulated at the protein-level in MGT_low_ and MGT_high_ tumors. For **c** and **d**, box hinges represent 1st and 3rd quartiles, and middle represents the median. The upper and lower whiskers extend from hinges up and down indicate the most extreme values that are within 1.5*IQR (interquartile range) of the respective hinge. The short horizontal lines represent the standard deviations

We next correlated the index with the expression of 20 potentially targetable immune checkpoints—that are currently in preclinical or clinical trial stages, and/or FDA-approved^22^ (Fig. 5a and Supplementary Data file S13). Unexpectedly, the data revealed strong correlations of TMI with B7-H3 expression in all (MMD) and nearly all (TCGA) tumor samples regardless of cancer types. Having observed mRNA-protein expression correlation using the processed proteomic data in TCGA BRCA cohort (Fig. 5b; Spearman’s rho test *r*_*s*_ = 0.38 and *P* = 4.19E−05), we found markedly higher levels of B7-H3 protein expression in TMI_high_ breast tumors (Fig. 5c). We thus speculate that the present combined quantitative analyses may help to unveil specific immune targets that could be selectively efficacious in a subset of tumors across diverse carcinomas.

### Promising predictive value for immunotherapy and other potential targets

We questioned if TMI could further be associated with innate PD-1 resistance signature (IPRES), including genes involved in immunosuppression, angiogenesis, monocyte and macrophage chemotaxis, and EMT,^21^ comparatively with other commercially available multi-gene tests (MGTs). Given that TMI signatures were derived from lung cancer, we chose lung cancer-derived prognostic MGTs and their predictive values for immunotherapy were assessed using lung cancer datasets (lung MMD and TCGA LUAD), with MGT-specific predefined cut-off thresholds for risk stratification (see Methods). The Myriad myplan^TM^ Lung Cancer and Pervenio^TM^ Lung RS tests are the only currently available prognostic MGTs for lung cancers, which comprise 31 cell cycle progression (CCP) genes and 11 cancer-related pathway genes, respectively. For each validation dataset, *IPRES-enriched* patients were identified as previously described^21^ (see Methods), and demonstrated significant differences in TMI, but not in other prognostic indices, with the exception of Pervenio^TM^ in the MMD cohort (Fig. 5d).

To identify potential therapeutic targets for both TMI_low_ and TMI_high_ groups, we performed differential proteomic analyses comparing the two groups and investigated if there would be any overlapping targets identified by other commercially available MGTs. Of note, as matched proteomic data were available only for breast cancer (TCGA BRCA), we analyzed two breast cancer MGTs (Oncotype DX and MammaPrint) in addition to the two lung cancer MGTs (Fig. 5e; Supplementary Data file S14; see Methods). Interestingly, while both breast and lung cancer MGTs demonstrated several differentially expressed proteins between the two groups, we observed new potential targets that were exclusive to either lung or breast cancers (Fig. 5f; Supplementary Data file S15). Further, a total of 4 (CASP14, CENPI, PAX9, and TDRD12) and 12 (FAM174A, UBXN10, BBS5, C9orf40, CACNA2D2, TFF1, UBXN10, RIPK4, CALHM6, WFDC2, NAGPA) proteins were consistently enriched in MGT_high_ and MGT_low_ tumors in TCGA BRCA cohort, respectively (Fig. 5g).

## Discussion

This work is based on multi-platform evaluation of TMI at multiple molecular levels across 11 major cancer types. By curating a total of 8836 patient-derived tumor and tumor-free samples, we generated 11 cancer type-specific MMDs annotated with clinical features and deposited the data at ArrayExpress (see Data availability). This approach minimizes biased selection of validation cohorts and allows parallel analyses with other high-throughput data sources such as TCGA.

Considering that the index was computed under the assumptions of each gene having concordant regulation as observed in lung cancer, it is noteworthy that tumors with highly dynamic and constantly remodeling ECM could have ubiquitously altered expression of matrisome genes across genetically and phenotypically diverse epithelial tumors. The data may imply the presence of shared signal transduction pathways activated across these selected tumors, such as recently reported TGFβ or HIF1α/VEGF pathways, in controlling ECM balance or cell-intrinsic mechanism regulating the expression of set of ECM genes contributing to tumor development.^23^

The functions of TMI matrisome genes, including secreted factors and metalloproteinases, in forming the *pre-metastatic niche* at specific target site remain poorly defined despite their well-established role in local matrix degradation and regulation.^24,25^ Exceptions are MMP1, which induces vascular permeability and mediates breast cancer metastasis to the lung,^24,26^ and S100 proteins, which generate pro-inflammatory phenotypes and recruit non-resident, bone marrow-derived cells.^24,27,28^ Intriguingly, our survival analyses revealed that TMI was correlated with a trophism for metastasis development to the lungs, but not to the bones in breast cancer patients, albeit in a subset of studies (Fig. 2b). This may suggest that the TMI signatures could inform on the tumor promoting microenvironment that facilitates metastasis to respective tissue organs. Through a matched proteomic data analysis, we further identified a small number of specific genes consistently highly expressed at protein level in both risk groups classified by TMI and other commercially available MGTs. Although their expression levels and prognostic values in malignant tumors remain inconclusive, high expression of CASP14^29^ and CENPI^30^ have been associated with poor prognosis in breast cancer, consistent with our observations in TMI_high_ patient group.

In addition to close associations with patient survival, we provide a comprehensive overview of commonly deregulated matrisome pattern in the context of tumor genotypes, molecular phenotypes, and immune response. Particularly, our combined quantitative analyses of matched proteomes revealed significant enrichment of MAGEA3 and CD8+ T cell signatures in TMI_low_ tumors. MAGEA3 is a cancer-germline gene highly expressed in various carcinomas, but the gene is silent in normal adult tissues, except for the testis and placenta.^31^ Independent of tumor burden, the prognostic value of MAGEA3-reactive CD8+ T cells for overall survival was reported in esophageal squamous cell carcinoma.^20^ Further, the study observed elevated expansion of functional MAGEA3-specific CD8+ T cells both in vitro and in vivo upon anti-PD-1 treatment.^20^ It is thus tempting to speculate that the genes included in the TMI signature might have functional roles for immune surveillance in the tumor microenvironment, given that MAGEA3 was the second most differentially expressed gene and anti-PD-1 therapy responder signature was highly enriched in TMI_low_ tumors.

To assess if the present approach could provide new indications for immunotherapy, we next associated TMI directly with the previously reported predictive biomarkers for ICI-based treatments, such as TMB, PD-L1 expression, TIL density, peripheral blood markers and other immune gene signatures.^9^ Of 20 targetable immune checkpoints, B7-H3 was the only gene with higher protein expression in TMI_high_ tumors in TCGA BRCA cohort. Consistent with our findings, increasing evidence suggests B7-H3 as a negative predictor of patient outcomes in solid tumors, including breast cancer.^32–35^ Further, its functional contribution to T cell inhibition and immune evasion is increasingly being recognized, making the molecule an appealing target as a novel immunotherapeutic drug.^36,37^ Beyond its role as an immune regulator, the functional impact of B7-H3 on cancer progression, including migration,^38^ invasion,^39^ angiogenesis,^40^ and gene regulation via epigenetic modifiers have also been suggested in a variety of cancers.^36^ Here we demonstrate a potential clinical utility of TMI as a pan-tumor predictive biomarker to identify a subset of patients who may benefit from anti-B7-H3 treatment. This concept could be validated using the same predefined cut-off for TMI-based risk stratification in a prospective clinical trial of this potential drug target.

Nonetheless, a clinically important finding in our study relates to the relative predictive potential of the respective immune response signatures in specific cancer types. For example, in lung cancer, the IPRES signatures were enriched in TMI_high_ tumors, which had higher TMB and no significant change in PD-L1 expression level as well as CD8+ T cell density relative to TMI_low_ tumors. These data point toward a closer association of IPRES with matrisomal abnormalities than with other previously reported signatures predictive of clinical response to ICI-based immunotherapy in this subset of tumors. As evidenced by recent clinical studies, tumors with high PD-L1 expression might not necessarily have low TIL density, or vice versa – for which either PD-L1 level or TIL abundance alone might not serve as an ideal predictive biomarker.^9,41,42^ However, more recent evidence do suggest that specific biomarkers such as TMB harbor a broader clinical utility to predict immunotherapy response across tumor types.^14,15^ Taken together, we propose the design of combinatorial approaches whereby tumor agnostic biomarkers are integrated with tumor-specific molecular indices, so as to optimize the accuracy of predicting immunotherapy response in specific cancer types.

## Methods

### Data preprocessing and MMD generation

This article and the accompanying data descriptor were previously published as preprints.^43,44^ Ethics approval was not required. Public datasets analyzed in this study are summarized in Supplementary Data file S1. Preprocessing methods, number of patient sample, platform assayed, and genes included in the computation of TMI are recorded in Supplementary Data file S2. Raw data of independent studies were RMA-normalized using the *affy* package^45^ or preprocessed- or author-defined normalized-data were used (Supplementary Data file S2). Most were assayed with the full 29-gene platform (i.e., Affymetrix Human Genome U133 Plus 2.0 Array). Probes having maximum expression values were collapsed to the genes for subsequent index scoring. Comprising a total of 8,386 samples, 95 independent GEO datasets (http://www.ncbi.nlm.nih.gov/geo), where raw data profiled on GPL570 platform were preprocessed, merged, and ComBat-adjusted (batch-effect removed) using the *inSilicoMerging* package^46^ based on cancer type, as previously done for lung MMD.^7^

### TCGA datasets

Using *TCGA-Assembler* package,^47^ the Cancer Genome Atlas (TCGA) data of 11 epithelial cancer types were collected, processed, and annotated with clinical parameters (Supplementary Data files S1 and S2). Due to lack of normal tissue samples, OV and SKCM cohorts, representing ovarian and melanoma cancers respectively, were excluded in differential expression analyses. *TCGA-Assembler* R package^47^ was used to extract normalized RPKM count values. In each dataset, we excluded genes without minimum 1 counts per million (cpm) or RPMK value in <20% of total number of samples were excluded using *edgeR* package.^48^ These filtered genes were normalized by Trimmed Mean of M-values (TMM) and were subjected to the *voom* function in the *limma* package^49^ for further analyses.

### *Generic* TMI signature

We extracted the ranking of 29 TMI genes (Table S3) and visualized their positions in each DEG list from each cancer-specific TCGA cohort using circular plots generated via the *circlize* package.^50^ To further investigate the extent to which TMI genes exhibit greater degree of differential expression variation among 29 lung-specific TMI genes across all tumors, we derived a *generic* TMI signature based on a weight computed for each TMI gene using the following equation (1), which was slightly modified from equation that was used to derive *generic* EMT signature in a previous study^51^:

1 $${\mathrm{weight}}\,(g) = \mathop {\sum}\nolimits_{{\mathrm{d} = 1}}^{D} {{\mathrm{log}2}\left({fc_{gd}} \right)} \times \frac{2.0}{(P_{gd} + {1.0)}} \times \frac{n_{d}}{\mathop {\sum }\nolimits_{\mathrm{i} = 1}^{\mathrm{D}} {n_i}}$$

where D is the total number of diseases (*D* = 7 in this study; prostate, renal, gastric, colorectal, breast, liver, and bladder cancer), *fc*_*gd*_ and *P*_*gd*_ are the fold-change and adjusted *P* value of the TMI gene, g, of disease, *d*, and *n*_*d*_ is the number of patient samples in each TCGA cohort (Supplementary Data file S3). As not all 29 TMI genes were present in the final cancer-specific DEG list due to preprocessing, each gene was computed with different number of total *n*_*i*_ for the weight. *Generic* TMI signature was then derived from 17 TMI genes having a weighted sum >3.90 and further visualized for their position in the ranked DEG list in a circular plot (Fig. 1b). SFTPC gene was not present in the final DEG list of all TCGA cohorts, except for lung cancer cohort which was not included in the weight computation.

### Prognostic index computation and patient stratification

Our 29-gene TMI signature comprise core and non-core matrisome components, which have previously been constructed using bioinformatics approaches^7,8^; based on the MatrisomeDB database,^4^ TMI’s core-matrisome molecules include “collagens” (COL11A1, COL10A1, COL6A6), “ECM glycoproteins” (SPP1, CTHRC1, TNNC1, ABI3BP, PCOLCE2), and “proteoglycan” (OGN); non-core matrisome molecules include “ECM regulators” (MMP12, MMP1, ADAMTS5), “ECM-affiliated proteins” (GREM1, SFTPC, SFTPA2, SFTPD, FCN3), “secreted factors” (S100A2, CXCL13, WIF1, CHRDL1, CXCL2, IL6, HHIP, S100A12), and other ECM-related components (LPL, CPB2, MAMDC2, CD36). Expression level of each 29 TMI gene was extracted from collapsed normalized data, and was multiplied by predefined Cox regression coefficient to compute the sum of these values, or the final index. As previously described,^8^ preprocessing filtered out different number of genes depending on TCGA dataset (Supplementary Data file S2), and cut-off index was determined using the Cutoff Finder algorithm^52^ to stratify each patient cohort into TMI_low_ and TMI_high_ groups. For four other MGTs analyzed in this study, the descriptive list of gene signatures, computation method, predefined threshold used for patient stratification and the associated references are stated in Supplementary Data file S14.

### Diagnostic performance of TMI

Complete lists of the patient ID and respective personalized TMI from both tumor and tumor-free samples across microarray and RNA-seq platforms are recorded in Supplementary Data files S4 and S5, respectively. TMI of tumor and normal groups were compared using the Mann–Whitney–Wilcoxon test. To further statistically evaluate the diagnostic accuracy of the TMI in deciding the presence of the disease, we computed the area under the receiver operating characteristic (ROC) curve (AUC), sensitivity, and specificity with the best threshold (Table S4) using the *pROC* package.^53^ All ROC curves generated were subjected to binormal smoothing for illustration (Fig. 1c).

### TMI spectrum quantification

We analyzed the Expression Project for Oncology (expO) data provided by the International Genomics Consortium (IGC, USA, www.intgen.com) for TMI spectrum quantification, for tumor samples procured and processed under standard conditions, resulting in minimal non-biological variations across multiple cancer types. All tumor specimens annotated with prostate, lung, renal, liver, gastric, breast, ovarian, pancreatic, colorectal, and bladder cancer from the expO dataset were included in the spectrum quantification (Fig. S1 and Supplementary Data file S6).

### Survival analyses

A statistical summary of datasets used in survival analyses is recorded in Supplementary Data file S7. Kaplan–Meier (KM) survival curves were derived for OS and DSS and other multiple endpoints using the *survival* package in R (http://CRAN.R-project.org/package = survival). Clinical data for TCGA datasets were collected via the embedded *DownloadBiospecimenClinicalData* function in the *TCGA-Assembler* package.^47^ Multivariate Cox regression analyses were performed to adjust confounding factors including age, race, gender, pT, pN, and pM status (Supplementary Data file S8). For all cancer types, patients with available survival data and TMI were all included in the KM analyses.

### Molecular subtyping in breast cancer

Of analyzed datasets, five datasets (BRCA, GSE20711, GSE21653, GSE19615, and GSE50567) were annotated with either ER, PR, and HER2 status or predefined molecular subtypes of breast cancers (normal breast-like, luminal A, luminal B, HER2 positive, and basal-like). Tumors harboring positive status of ER and/or negative status of HER2 were classified as luminal cancers while the basal-like tumors were defined as ER-, PR-, and HER2-negative cancers (Supplementary Data file S10).

### Total mutational burden

The mutational load of TCGA tumor samples across nine epithelial cancer types, for which TMI was previously computed, was obtained from the National Cancer Institute GDC Data Portal (http://portal.gdc.cancer.gov/projects/). The portal defines the mutational load as the total number of simple somatic mutations. Only tumors harboring at least one mutation were included and log 10 transformed to compute Spearman correlations between TMI and mutational load. A descriptive statistics including the number of patient samples available for both TMI gene expression and mutational load data, cut-off points used for patient stratification, and key outputs of Spearman correlation tests in each dataset is shown in Supplementary Data file S11.

### IPRES and GSVA

Gene signatures previously associated with resistance to PD-1 immunotherapy, termed Innate PD-1 RESistance (IPRES), were obtained from Broad MSigDB (http://software.broadinstitute.org/gsea/msigdb) and Supplementary Data provided by the original publication.^21^ The gene set variation analysis (GSVA) scores of the signatures were computed for each MMD and TCGA patient sample. GSVA scores were transformed to z-scores, and were correlated with TMI. As previously described,^21^ a cut-off of > 0.35 was applied to the mean z-score for a patient to be determined as *IPRES-enriched*. As these signatures were derived from non-responder (resistant) tumors, we further defined *responder signatures* as 161 highly expressed genes (log FC > 2 and Mann–Whitney *P* < 0.1) in responders compared to non-responders using the list of 693 DE genes provided by the original work.^21^

### CIBERSORT

The estimated fraction of individual immune cell types was computed using the beta version of CIBERSORT (http://cibersort.stanford.edu/). For any given sample, we calculated Spearman correlation between TMI and relative abundance of each immune cell type using our 11 generated MMDs and 11 TCGA cohorts (LUAD, OV, SKCM, BLCA, LIHC, BRCA, COAD, STAD, KIRC, PRAD, and PAAD). As our MMDs of breast, colorectal, and lung cancer exceeded maximum load capacity (500 MB), 1000 tumors were randomly selected for input data files. We selected LM22 (22 immune cell types) for signature gene file, 100 for permutations, and disabled quantile normalization for all runs.

### Quantitative proteomic analysis

Relative protein abundance data of 10,625 protein-coding genes were generated by the National Cancer Institute Clinical Proteomic Tumor Analysis Consortium (CPTAC; https://cptc-xfer.uis.georgetown.edu/publicData/Phase_II_Data/TCGA_Breast_Cancer/). Log ratios (base 2), representing relative abundance of each sample compared to the pooled reference sample, were obtained from *TCGA_Breast_BI_Proteome.itraq.tsv* for a total of 108 TCGA BRCA breast cancer samples, for which TMI was previously computed.

### Differential expression analysis and heatmaps

R/Bioconductor *limma* package^49^ was used to assess differential protein expression between two patient groups classified by five MGTs. FC > 1.5 or FC < −1.5 and *limma P* < 0.05 were applied to determine DE proteins. A descriptive list of all DEGs at the protein level found in both groups is provided in Supplementary Data file S15. Heatmaps used in this study were generated using Morpheus (http://software.broadinstitute.org/morpheus/).

## Supplementary information

Supplementary Information

Data file S1

Data file S2

Data file S3

Data file S4

Data file S5

Data file S6

Data file S7

Data file S8

Data file S9

Data file S10

Data file S11

Data file S12

Data file S13

Data file S14

Data file S15

## Data Availability

Our generated cancer type-specific MMDs are available at ArrayExpress under accession codes E-MTAB-6690 (pancreatic cancer), E-MTAB-6691 (ovarian cancer), E-MTAB-6692 (renal cancer), E-MTAB-6693 (gastric cancer), E-MTAB-6694 (prostate cancer), E-MTAB-6695 (liver cancer), E-MTAB-6696 (bladder cancer), E-MTAB-6697 (melanoma cancer), E-MTAB-6698 (colorectal cancer), E-MTAB-6699 (lung cancer), and E-MTAB-6703 (breast cancer). The accession codes of all public datasets analyzed in this study are listed in Supplementary Data file S1.

## Supplementary information

**Supplementary Information** accompanies the paper on the *npj Precision Oncology* website (10.1038/s41698-019-0087-0).

## Code availability

The R codes used to preprocess, merge and batch-effect correct datasets for generation of all 11 MMDs can be found in Figshare (10.6084/m9.figshare.7878086).

## References

[1] Hynes RO (2009). The extracellular matrix: not just pretty fibrils. Science.

[2] Bateman JF, Boot-Handford RP, Lamande SR (2009). Genetic diseases of connective tissues: cellular and extracellular effects of ECM mutations. Nat. Rev. Genet..

[3] Lu P, Weaver VM, Werb Z (2012). The extracellular matrix: a dynamic niche in cancer progression. J. Cell Biol..

[4] Naba A (2016). The extracellular matrix: tools and insights for the “omics” era. Matrix Biol..

[5] Hynes RO, Naba A (2012). Overview of the matrisome–an inventory of extracellular matrix constituents and functions. Cold Spring Harb. Perspect. Biol..

[6] Naba A (2012). The matrisome: in silico definition and in vivo characterization by proteomics of normal and tumor extracellular matrices. Mol. Cell Proteom..

[7] Lim SB, Tan SJ, Lim W-T, Lim CT (2018). A merged lung cancer transcriptome dataset for clinical predictive modeling. Sci. Data.

[8] Lim SB, Tan SJ, Lim WT, Lim CT (2017). An extracellular matrix-related prognostic and predictive indicator for early-stage non-small cell lung cancer. Nat. Commun..

[9] Gibney GT, Weiner LM, Atkins MB (2016). Predictive biomarkers for checkpoint inhibitor-based immunotherapy. Lancet Oncol..

[10] Uhlen, M. et al. A pathology atlas of the human cancer transcriptome. *Science* **357**, eaan2507, 10.1126/science.aan2507 (2017). 10.1126/science.aan2507 28818916

[11] Hoadley KA (2014). Multiplatform analysis of 12 cancer types reveals molecular classification within and across tissues of origin. Cell.

[12] Tobin NP (2015). Molecular subtype and tumor characteristics of breast cancer metastases as assessed by gene expression significantly influence patient post-relapse survival. Ann. Oncol..

[13] Carey LA (2006). Race, breast cancer subtypes, and survival in the Carolina Breast Cancer Study. JAMA.

[14] Miao D (2018). Genomic correlates of response to immune checkpoint blockade in microsatellite-stable solid tumors. Nat. Genet..

[15] Samstein RM (2019). Tumor mutational load predicts survival after immunotherapy across multiple cancer types. Nat. Genet..

[16] Sade-Feldman M (2018). Defining T cell states associated with response to checkpoint immunotherapy in melanoma. Cell.

[17] Tumeh PC (2014). PD-1 blockade induces responses by inhibiting adaptive immune resistance. Nature.

[18] Chen PL (2016). Analysis of immune signatures in longitudinal tumor samples yields insight into biomarkers of response and mechanisms of resistance to immune checkpoint blockade. Cancer Disco..

[19] Huang AC (2017). T-cell invigoration to tumour burden ratio associated with anti-PD-1 response. Nature.

[20] Chen X (2018). Dual TGF-beta and PD-1 blockade synergistically enhances MAGE-A3-specific CD8(+) T cell response in esophageal squamous cell carcinoma. Int. J. Cancer.

[21] Hugo W (2016). Genomic and transcriptomic features of response to anti-PD-1 therapy in metastatic melanoma. Cell.

[22] Mak MP (2016). A patient-derived, pan-cancer EMT signature identifies global molecular alterations and immune target enrichment following epithelial-to-mesenchymal transition. Clin. Cancer Res.

[23] Naba A, Clauser KR, Lamar JM, Carr SA, Hynes RO (2014). Extracellular matrix signatures of human mammary carcinoma identify novel metastasis promoters. Elife.

[24] Peinado H (2017). Pre-metastatic niches: organ-specific homes for metastases. Nat. Rev. Cancer.

[25] Lu X (2009). ADAMTS1 and MMP1 proteolytically engage EGF-like ligands in an osteolytic signaling cascade for bone metastasis. Genes Dev..

[26] Minn AJ (2005). Genes that mediate breast cancer metastasis to lung. Nature.

[27] Gupta GP (2007). Mediators of vascular remodelling co-opted for sequential steps in lung metastasis. Nature.

[28] Peinado H (2012). Melanoma exosomes educate bone marrow progenitor cells toward a pro-metastatic phenotype through MET. Nat. Med..

[29] Handa T (2017). Caspase14 expression is associated with triple negative phenotypes and cancer stem cell marker expression in breast cancer patients. J. Surg. Oncol..

[30] Thangavelu PU (2017). Overexpression of the E2F target gene CENPI promotes chromosome instability and predicts poor prognosis in estrogen receptor-positive breast cancer. Oncotarget.

[31] Peled N, Oton AB, Hirsch FR, Bunn P (2009). MAGE A3 antigen-specific cancer immunotherapeutic. Immunotherapy.

[32] Maeda N (2014). Expression of B7-H3, a potential factor of tumor immune evasion in combination with the number of regulatory T cells, affects against recurrence-free survival in breast cancer patients. Ann. Surg. Oncol..

[33] Dong P, Xiong Y, Yue J, Hanley SJB, Watari H (2018). B7H3 as a promoter of metastasis and promising therapeutic target. Front. Oncol..

[34] Zhang X (2017). Prognostic value of B7-H3 expression in patients with solid tumors: a meta-analysis. Oncotarget.

[35] Arigami T (2010). B7-h3 ligand expression by primary breast cancer and associated with regional nodal metastasis. Ann. Surg..

[36] Castellanos JR (2017). B7-H3 role in the immune landscape of cancer. Am. J. Clin. Exp. Immunol..

[37] Pardoll DM (2012). The blockade of immune checkpoints in cancer immunotherapy. Nat. Rev. Cancer.

[38] Chen YW, Tekle C, Fodstad O (2008). The immunoregulatory protein human B7H3 is a tumor-associated antigen that regulates tumor cell migration and invasion. Curr. Cancer Drug Targets.

[39] Tekle C (2012). B7-H3 contributes to the metastatic capacity of melanoma cells by modulation of known metastasis-associated genes. Int. J. Cancer.

[40] Ricci-Vitiani L (2010). Tumour vascularization via endothelial differentiation of glioblastoma stem-like cells. Nature.

[41] Taube JM (2014). Association of PD-1, PD-1 ligands, and other features of the tumor immune microenvironment with response to anti-PD-1 therapy. Clin. Cancer Res..

[42] Kluger HM (2015). Characterization of PD-L1 expression and associated T-cell infiltrates in metastatic melanoma samples from variable anatomic sites. Clin. Cancer Res..

[43] Lim, S. B., Tan, S. J., Lim, W.-T. & Lim, C. T. Cross-platform meta-analysis reveals common matrisome variation associated with tumor genotypes and immunophenotypes in human cancers. Preprint at 10.1101/353698 (2018).

[44] Lim, S. B., Tan, S. J., Lim, W.-T. & Lim, C. T. Compendiums of cancer transcriptome for machine learning applications. Preprint at 10.1101/353706 (2018).

[45] Gautier L, Cope L, Bolstad BM, Irizarry RA (2004). Affy–analysis of Affymetrix GeneChip data at the probe level. Bioinformatics.

[46] Taminau J (2012). Unlocking the potential of publicly available microarray data using inSilicoDb and inSilicoMerging R/Bioconductor packages. BMC Bioinf..

[47] Zhu Y, Qiu P, Ji Y (2014). TCGA-assembler: open-source software for retrieving and processing TCGA data. Nat. Methods.

[48] Robinson MD, McCarthy DJ, Smyth GK (2010). edgeR: a Bioconductor package for differential expression analysis of digital gene expression data. Bioinformatics.

[49] Ritchie ME (2015). limma powers differential expression analyses for RNA-sequencing and microarray studies. Nucleic Acids Res..

[50] Gu Z, Gu L, Eils R, Schlesner M, Brors B (2014). circlize Implements and enhances circular visualization in R. Bioinformatics.

[51] Tan TZ (2014). Epithelial-mesenchymal transition spectrum quantification and its efficacy in deciphering survival and drug responses of cancer patients. EMBO Mol. Med..

[52] Budczies J (2012). Cutoff Finder: a comprehensive and straightforward Web application enabling rapid biomarker cutoff optimization. PLoS ONE.

[53] Robin X (2011). pROC: an open-source package for R and S+to analyze and compare ROC curves. BMC Bioinf..

