# Supplementary material for: Pan-cancer analysis connects tumor matrisome to immune response
Source: NPJ Precis Oncol. 2019 May 22;3:15. doi: 10.1038/s41698-019-0087-0 (PMC6531473; doi:10.1038/s41698-019-0087-0)
Supplement: Supplementary file 1 — Supplementary Information [file 41698_2019_87_MOESM1_ESM.pdf]

## Supplementary Figures

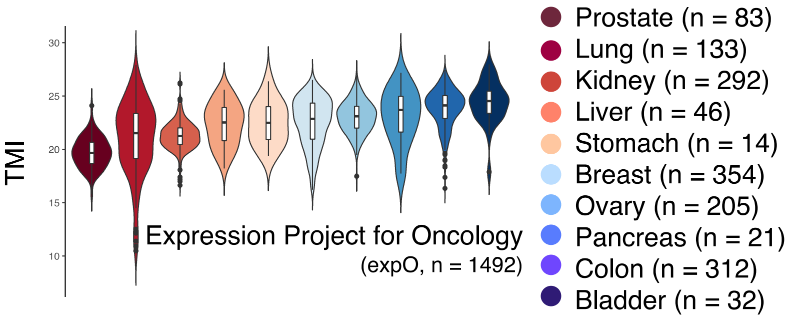


**Fig. S1.** Inter-tumor variation of matrisome abnormalities across 11 cancer types using an independent data source (see Methods).

## Supplementary Tables

**Table S1.** Intra-platform genome-wide DE analysis (microarray): Spearman's correlation coefficients across 11 cancer-specific meta-datasets.

| MMD | Lung | Pancreas | Prostate | Kidney | Stomach | Colon | Ovary | Breast | Liver | Bladder | Melanoma |
|---|---|---|---|---|---|---|---|---|---|---|---|
| Spearman correlation coefficient with lung MMD | 1.000 | -0.002 | 0.311 | -0.027 | 0.161 | 0.315 | 0.397 | 0.482 | 0.250 | 0.300 | 0.150 |
| # of total samples | 1621 | 178 | 237 | 323 | 737 | 1514 | 647 | 2302 | 401 | 212 | 214 |
| # of tumor samples | 1474 | 108 | 121 | 219 | 691 | 1393 | 593 | 2088 | 244 | 161 | 194 |
| # of normal samples | 147 | 70 | 116 | 104 | 46 | 121 | 54 | 214 | 157 | 51 | 20 |
| # of common genes with lung MMD | - | 20546 | 20546 | 20546 | 20546 | 20546 | 20546 | 20546 | 20546 | 20546 | 20546 |

**Table S2.** Intra-platform genome-wide DE analysis (RNA-seq): Spearman's correlation coefficients across 12 TCGA cohorts.

| TCGA | LUAD | LUSC | PAAD | PRAD | KICH | KIRC | KIRP | STAD | COAD | BRCA | LIHC | BLCA |
|---|---|---|---|---|---|---|---|---|---|---|---|---|
| Spearman correlation coefficient with LUAD | 1.000 | 0.810 | 0.380 | 0.442 | 0.168 | 0.107 | 0.325 | 0.453 | 0.508 | 0.595 | 0.428 | 0.644 |
| Spearman correlation coefficient with LUSC | 0.810 | 1.000 | 0.347 | 0.331 | 0.189 | 0.058 | 0.255 | 0.380 | 0.522 | 0.503 | 0.415 | 0.603 |
| # of total samples | 574 | 553 | 182 | 549 | 90 | 606 | 323 | 450 | 327 | 1207 | 421 | 427 |
| # of tumor samples | 515 | 502 | 178 | 482 | 65 | 533 | 291 | 415 | 286 | 1094 | 371 | 408 |
| # of normal samples | 59 | 51 | 4 | 67 | 25 | 73 | 32 | 35 | 41 | 113 | 50 | 19 |
| # of common genes with LUAD | - | 14964 | 14927 | 14471 | 14438 | 14576 | 14542 | 14805 | 14380 | 14738 | 13687 | 14627 |
| # of common genes with LUSC | 14964 | - | 14848 | 14429 | 14348 | 14492 | 14438 | 14768 | 14479 | 14689 | 14656 | 14756 |

**Table S3.** Ranking of TMI genes based on their DE in tumors compared to normal tissues.

| | Rank | LUAD | PRAD | KIRP | STAD | COAD | BRCA | LIHC | BLCA |
|---|---|---|---|---|---|---|---|---|---|
| | # of Samples | 574 | 549 | 323 | 450 | 327 | 1207 | 421 | 427 |
| | # of Genes | 15404 | 14976 | 15115 | 15418 | 14785 | 15165 | 14138 | 15033 |
| 29 TMI genes | **ABI3BP** | 416 | 2580 | 4982 | 509 | 253 | 629 | 1046 | 112 |
| | **ADAMTS8** | 68 | 3365 | 1612 | 1001 | 1216 | 1353 | NA | 119 |
| | **CD36** | 184 | 1551 | 2534 | 820 | 400 | 56 | 9246 | 1480 |
| | **CHRDL1** | 349 | 189 | 237 | 113 | 171 | 33 | NA | 18 |
| | **COL10A1** | 64 | 14 | NA | 5 | 30 | 4 | 8490 | 77 |
| | **COL11A1** | 11 | NA | NA | 7 | 29 | 16 | NA | 79 |
| | **COL6A6** | 150 | NA | NA | NA | NA | 113 | NA | NA |
| | CPB2 | 22 | NA | NA | NA | NA | NA | 1578 | NA |
| | **CTHRC1** | 167 | 695 | 3445 | 171 | 408 | 710 | 338 | 4290 |
| | CXCL13 | 181 | 301 | NA | 1469 | 1112 | 815 | 1465 | 6273 |
| | CXCL2 | 940 | 3763 | 14762 | 6583 | 1435 | 72 | 627 | 510 |
| | FCN3 | 49 | NA | 438 | 3797 | 1949 | 5808 | 20 | 4916 |
| | GREM1 | 93 | 14919 | 652 | 6255 | 14261 | 3792 | 14044 | 12599 |
| | **HHIP** | 56 | 5190 | 1940 | 2474 | 776 | NA | 27 | 259 |
| | **IL6** | 851 | 3106 | 962 | 4605 | 4343 | 415 | NA | 92 |
| | LPL | 372 | 7283 | 565 | 8310 | 7682 | 64 | 809 | 4194 |
| | **MAMDC2** | 210 | 518 | 1735 | 36 | 46 | 156 | NA | 106 |
| | **MMP1** | 60 | 7543 | 2316 | 164 | 306 | 334 | 1334 | 589 |
| | MMP12 | 101 | 1570 | NA | 172 | 2883 | 2538 | 662 | 3107 |
| | **OGN** | 287 | 1390 | 110 | 505 | 60 | 181 | 1742 | 6 |
| | **PCOLCE2** | 307 | 4058 | 425 | 257 | 131 | 39 | 8518 | 55 |
| | S100A12 | NA | NA | NA | 441 | NA | NA | NA | 14975 |
| | S100A2 | 1073 | 1327 | 786 | 4694 | 233 | 5691 | NA | 2109 |
| | SFTPA2 | 52 | 418 | NA | 4405 | 1633 | 10775 | NA | 4915 |
| | SFTPC | 1 | NA | NA | NA | NA | NA | NA | NA |
| | SFTPD | 154 | 13261 | 1933 | NA | NA | 6618 | NA | NA |
| | **SPP1** | 61 | 2353 | 2320 | 166 | 613 | 1114 | 1234 | 389 |
| | TNNC1 | 75 | 908 | 210 | 705 | 2113 | NA | 2449 | 11768 |
| | **WIF1** | 23 | 52 | NA | NA | 14307 | 36 | NA | NA |

**Bold:** *Generic* TMI signature

**Table S4.** Cutoff TMI, AUC, specificity, and sensitivity across all 11 cancer types.

| GEO meta-datasets | Lung | Pancreas | Prostate | Renal | Stomach | Colon | Ovary | Breast | Liver | Bladder | Melanoma |
|---|---|---|---|---|---|---|---|---|---|---|---|
| Cutoff | 16.309 | 22.755 | 16.902 | 20.761 | 22.418 | 22.754 | 23.202 | 23.215 | 19.630 | 23.381 | 20.148 |
| AUC (%) | 94.6 | 87.5 | 77.9 | 81.2 | 77.7 | 86.4 | 80.6 | 80.7 | 82.5 | 63.1 | 91.8 |
| Specificity (%) | 95.2 | 91.4 | 75.86 | 83.65 | 82.61 | 80.99 | 75.93 | 81.31 | 76.64 | 82.35 | 90 |
| Sensitivity (%) | 83.4 | 79.6 | 68.6 | 71.23 | 73.37 | 87.01 | 72.68 | 71.07 | 76.89 | 46.58 | 88.14 |
| Number of samples | 1621 | 178 | 237 | 323 | 737 | 1514 | 647 | 2302 | 401 | 212 | 214 |
| Tumor N | 1474 | 108 | 121 | 219 | 691 | 1393 | 593 | 2088 | 244 | 161 | 194 |
| Normal N | 147 | 70 | 116 | 104 | 46 | 121 | 54 | 214 | 157 | 51 | 20 |

| TCGA data sets | LUAD | LUSC | PAAD | PRAD | KICH | KIRC | KIRP | STAD | COAD | | BRCA | LIHC | BLCA | |
|---|---|---|---|---|---|---|---|---|---|---|---|---|---|---|
| Cutoff | 15.306 | 19.818 | 31.940 | 23.463 | 28.276 | 26.945 | 27.233 | 27.134 | 27.437 | | 23.652 | 29.186 | 27.505 | |
| AUC (%) | 99.9 | 99.9 | 62.5 | 78.6 | 98.6 | 90.4 | 99.2 | 95.4 | 99.9 | | 99.9 | 81 | 99.5 | |
| Specificity (%) | 100 | 100 | 100 | 71.6 | 100 | 89 | 96.9 | 94.3 | 100 | NA | 100 | 90 | 100 | NA |
| Sensitivity (%) | 99.6 | 99.4 | 33.1 | 83.4 | 95.4 | 80.1 | 95.5 | 86.3 | 98.6 | | 99.5 | 66.3 | 95.3 | |
| Number of samples | 574 | 553 | 182 | 549 | 90 | 606 | 323 | 450 | 327 | | 1207 | 421 | 427 | |
| Tumor N | 515 | 502 | 178 | 482 | 65 | 533 | 291 | 415 | 286 | | 1094 | 371 | 408 | |
| Normal N | 59 | 51 | 4 | 67 | 25 | 73 | 32 | 35 | 41 | | 113 | 50 | 19 | |

**Table S5.** Summary of TCGA cohorts and respective statistical significance in mutational load correlation analyses.

| Cancer Type | Patient N annotated with both TMI & mutational load | Patient N having at least > 1 mutation | Spearman's *r* | Spearman's *P* | Mann-Whitney-Wilcoxon test *P* | TMI cut-off score |
|---|---|---|---|---|---|---|
| Bladder (BLCA) | 408 | 408 | 0.1128619 | 0.02261 | 0.0293 | 33.18 |
| Breast (BRCA) | 1094 | 982 | 0.2289674 | 1.77E-14 | 1.54E-05 | 35.36 |
| Colon (COAD) | 284 | 278 | 0.3600599 | 4.05E-10 | 0.002953 | 37.56 |
| Kidney (KIRC) | 309 | 309 | 0.1322628 | 0.02003 | 0.1038 | 28.96 |
| Liver (LIHC) | 371 | 359 | 0.2066888 | 6.04E-05 | 0.06456 | 31.47 |
| Pancreas (PAAD) | 177 | 172 | 0.4797647 | 1.41E-11 | 1.69E-07 | 28.16 |
| Prostate (PRAD) | 482 | 479 | 0.4079654 | < 2.2e-16 | 0.0003141 | 28.7 |
| Stomach (STAD) | 415 | 410 | 0.4678014 | < 2.2e-16 | 6.23E-11 | 30.96 |
| Lung (LUAD) | 515 | 511 | 0.355204 | < 2.2e-16 | 0.0003308 | 34.16 |
